# Healthcare providers’ knowledge and attitudes about overdose prevention sites in Colorado

**DOI:** 10.1186/s12954-024-01066-y

**Published:** 2024-08-24

**Authors:** Emily Paz, Vahid Mashhouri, Mark E. Payton, Brian D. Schwartz, Rachel M.A. Linger

**Affiliations:** 1 College of Osteopathic Medicine, Rocky Vista University, Englewood, CO USA; 2 Biomedical Sciences Department, Rocky Vista University, Englewood, CO USA; 3 Medical Humanities Department, Rocky Vista University, Englewood, CO USA

**Keywords:** Health care facilities, Health professionals, Opioids, Overdose prevention, Advocacy, Substance use, Survey, Colorado, United States

## Abstract

**Background:**

Overdose prevention sites (OPS) are a harm reduction strategy that offer people who use drugs a variety of resources including but not limited to sterile supplies, linkage to healthcare resources, and intervention if an overdose occurs. OPS operate in over 120 countries and evidence has demonstrated they are an effective harm reduction strategy. Despite their success elsewhere, OPS remain federally illegal in the United States and thus there is limited research on their implementation and outcomes in the United States. This study aimed to identify Colorado healthcare providers’ knowledge and attitudes about OPS and determine if there is a correlation between healthcare providers with more knowledge about OPS having a more positive attitude about OPS.

**Methods:**

An electronic survey was distributed to healthcare providers in Colorado. Responses were collected in early 2022 and recorded on a 5-point Likert scale. Mean scores between 1 and 5 were calculated for each participant and analysis of variance methods were used to determine correlating demographic factors. A *p* value of ≤ 0.05 was used to determine statistical significance of all findings.

**Results:**

This study included 698 participants. A Pearson correlation analysis revealed a strong positive relationship (*r* = 0.76, *p* < 0.0001) between provider knowledge and attitudes about OPS. Emergency medicine providers scored the highest in mean knowledge and attitude scores in comparison to all other specialties. Respondents affiliated with a harm reduction center exhibited the highest mean knowledge and attitude scores. Mean knowledge and attitude scores generally rose with respondents’ increasing encounters with people who inject drugs in a typical workday, except when reaching nine or more encounters, where a sharp decline occurred.

**Conclusions:**

Our study highlights the importance of education, exposure to harm reduction strategies, and inter-specialty collaboration in shaping healthcare providers’ knowledge and attitudes about OPS. The positive correlation between providers’ knowledge and attitudes about OPS suggests that educating healthcare providers on harm reduction strategies, specifically OPS, may lead to reduced stigmatization of OPS among healthcare professionals.

**Supplementary Information:**

The online version contains supplementary material available at 10.1186/s12954-024-01066-y.

## Background

Synthetic opioids are involved in over 136 overdose deaths a day in the United States [1]. The CDC predicted 113,312 overdose deaths from August 2022 to August 2023 [2]. Colorado is no exception to this national trend; the Colorado Institute of Health reported a 54% increase in overdose deaths in 2020 from the previous year with an associated spike in fentanyl related overdose deaths [3].

Harm reduction supports people who use drugs (PWUD) by offering strategies and tools to promote health and prevent overdose related deaths, including access to sterile syringes and pipes, drug testing strips, naloxone, health education, infectious disease testing, and linkage to treatment of infectious diseases and substance use disorders. Overdose prevention sites (OPS), an evidence-based harm reduction strategy, offer PWUD a secure place with sterile supplies to self-administer pre-obtained drugs under the supervision of staff trained to recognize an overdose and intervene if it occurs.

Overdose prevention sites offer participants supplies and guidance for safer self-administration practices, education on blood borne illnesses and prevention, and linkage to appropriate healthcare and public services [4]. A systematic literature review of 75 articles on OPS found that all studies reported positive impact on individual participants: safer injection practices, decreased overdose related mortalities, and effective connection of participants with primary healthcare services. Further, the review did not report a single overdose death inside an OPS [5]. Similarly, an OPS operating in New York City (NYC) for over two years has not had an overdose related death within the facility [6]. A study of OPS participants in Catalonia, Spain found frequent attendees had a 61% lower risk of public drug use and sharing of needles, were more likely to dispose of used syringes properly, and twice as likely to access treatment services [7]. In a separate study, OPS implementation did not significantly increase rates of relapse among people in recovery for former injection drug use or significantly decrease cessation among current injection drug users [8].

Common themes of concern for communities with an OPS include conceivable increases in drug-related crime in the surrounding area, an increase in the number of local drug users, and the cost of implementation [5, 9]. After the implementation of an OPS in Australia, researchers found there was no increase in theft or robbery surrounding the OPS, no increase in drug-related loitering at the front of the OPS, and a small increase in loitering behind the OPS, which they concluded was not significant enough to create a public nuisance [10]. An updated report nearly ten years after the OPS began operating found no evidence of negative impacts on robbery, property crime, or drug offenses [11].

In addition to their lack of negative impact, OPS have been shown to positively impact the surrounding community. Data from an OPS operating in Canada found decreased public drug use and related litter as well as reduced crime and drug trafficking around OPS [5]. In 2017, researchers conducted a cost-benefit analysis of a potential OPS in San Francisco. Five potential outcomes were assessed that could result in healthcare cost savings and public health benefits and concluded that a single OPS facility with 13 booths would result in a net savings of 3.5 million dollars per year [12]. Similarly, a systematic review identified five studies out of Canada that evaluated the economic impacts of OPS and all concurred that OPS are cost effective [9]. Overall, these data indicate OPS are a cost effective harm reduction strategy that does not increase drug use or drug related crime in the surrounding communities.

In November 2021, New York City (NYC) responded to the dramatic increase in opioid overdose deaths by establishing two OPS, with over 1,500 overdose interventions as of March, 2024 [6]. These unsanctioned OPS rely on private funding and face the threat of shut down by federal and state officials [13]. A pilot OPS in Providence, Rhode Island (RI) is set to open in 2024, and would be the first state-authorized and operated OPS [14, 15]. The National Institute on Drug Abuse has funded a four year longitudinal study analyzing the impact of OPS in NYC and RI [16]. Recent data on the NYC sites have confirmed no increase in crime or disorder in the surrounding areas [17]. In Colorado, legislation was introduced in 2018 and 2023 to authorize implementation of OPS in Denver, but both ultimately lost [18, 19].

Because OPS are not federally sanctioned by the U.S. government there are few published studies on the impact of OPS in the U.S. One study of an unsanctioned OPS in the U.S. found that zero overdose deaths occurred during five years of operation despite over 10,000 injection events and 33 opioid overdoses, suggesting that OPS could reduce mortality from opioid overdose [20]. OPS have been extensively studied in Vancouver, Canada, and Sydney, Australia, where they have served as important harm reduction measures since 2003 and 2001, respectively. Over 120 OPS are operating across Canada, Australia, and Europe [21, 22]. The American Medical Association acknowledges the significant need for harm reduction implementation and the success of OPS in other countries [23, 24].

A study by the Harm Reduction Action Center of Colorado conducted in 2020 assessed healthcare providers attitudes towards caring for people who inject drugs (PWID); a higher score indicated a more positive attitude. Their survey included all healthcare workers and found that physicians scored the highest in comparison to other healthcare roles except for social workers. The vast majority of physicians agreed with the survey statement that it was their responsibility to make sure PWID are knowledgeable about harm reduction practices. The study also identified the most common barriers that prevent healthcare providers from caring for PWID and connecting them with harm reduction resources. They included limited provider knowledge, both about harm reduction and where services are available, perceiving PWID as adversarial, and not having the time to discuss harm reduction [25]. The data suggest that increasing provider knowledge would help mitigate these barriers.

It remains unknown whether healthcare providers’ knowledge and attitudes about harm reduction practices like OPS are correlated. This gap in knowledge inspired our hypothesis that a provider who is more knowledgeable about OPS will have a more positive attitude about OPS. To test this hypothesis, we created a survey to distribute to Colorado healthcare providers. This research is significant as healthcare providers are known to play a significant role in the implementation of harm reduction strategies. Research suggests providers can positively impact harm reduction efforts as has been previously seen with increasing naloxone prescriptions by equipping providers with the knowledge and tools to do so [26, 27]. The findings from this survey establish a baseline of knowledge and attitudes about OPS amongst Colorado healthcare providers. These results can be used to inform targeted strategies to improve harm reduction practices, specifically OPS implementation.

## Methods

After review and approval by the appropriate Institutional Review Board, an online survey was distributed to providers practicing in Colorado healthcare organizations using the authors’ personal networks, which included contacts at local and regional professional associations and health care systems. An informed consent was included at the beginning of the survey. Data were collected via Qualtrics. All responses were kept confidential and stored in a protected digital format. No personal identifying information was collected other than an option to provide an email address to be entered into a random drawing for a monetary incentive. The survey was open from January 18th, 2022 through March 21st, 2022. Inclusion criteria consisted of providers with credentials of DO, MD, PA, or NP currently licensed and practicing in the state of Colorado.

The 21-item survey consisted of three sections: demographic information (6 questions), providers’ evidence-based knowledge about OPS (7 statements) and providers’ attitudes about OPS (8 statements) (Supplemental Table 1). The statements within the providers’ knowledge and attitude sections were presented in random order. Survey statements assessing knowledge are based on evidence from previous research as summarized in the Introduction. Raw data were tabulated across a 5-point Likert scale. Agree and strongly agree were interpreted as being in support of the statement, while disagree and strongly disagree were collectively recorded as not in support of the statement. Statement number 6, “OPS are an effective way to connect PWID with other services including healthcare,” had the lowest number of responses. Those that did not respond to this statement were excluded from analysis.

Our hypothesis expected a more knowledgeable provider with a more positive attitude towards OPS to support statements 1, 3, 6, 7, 8, 9, 10, 11, 14, and 15. Statements 2, 4, 5, 12, and 13 are reverse coded, and a knowledgeable provider with a more positive attitude towards OPS was expected to oppose those statements. Responses congruent with our hypothesis were assigned higher numeric values (Supplemental Table 2). Questions without a response were omitted on a per participant basis and the mean score of questions answered was analyzed for each participant. Using this scoring system, each participant received a mean knowledge score and a mean attitude score between 1 and 5. A higher mean score indicates a participant is more knowledgeable and holds a more positive attitude about OPS. Mean score variances were analyzed for correlating demographic factors. An intra-instrument analysis was used to identify and eliminate any outliers that may skew the analysis.

## Results

**Fig. 1:**
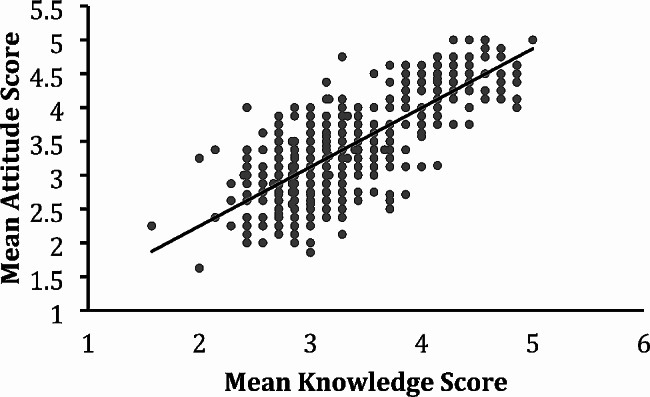
Correlation between Mean Knowledge and Attitude Scores of Providers. Closed circles represent individual participant scores (*n*=698) plotted as mean knowledge score on the x-axis and mean attitude score on the y-axis. The solid line was generated by a Pearson correlation analysis indicating a strong positive relationship (r = 0.76, *p* < 0.0001) between provider knowledge and attitude about OPS

Out of 735 recorded responses, 698 were included in our analysis representing a variety of specialties and demographics (Supplemental Table 3). An analysis using a Pearson Correlation Coefficient revealed a strong positive linear relationship (*r* = 0.76, *p* < 0.0001) between provider knowledge and attitude about OPS (Fig. 1).

Of the 698 responses, 274 identified as MDs, 187 as DOs, 182 as PAs, and 52 as NPs. Three participants did not identify their roles. No one provider role scored significantly higher or lower than the rest in either knowledge or attitude (*p* = 0.14 and 0.14, respectively), meaning all provider types surveyed were comparable in knowledge and attitudes about OPS (Supplemental Figure S1).

**Fig. 2:**
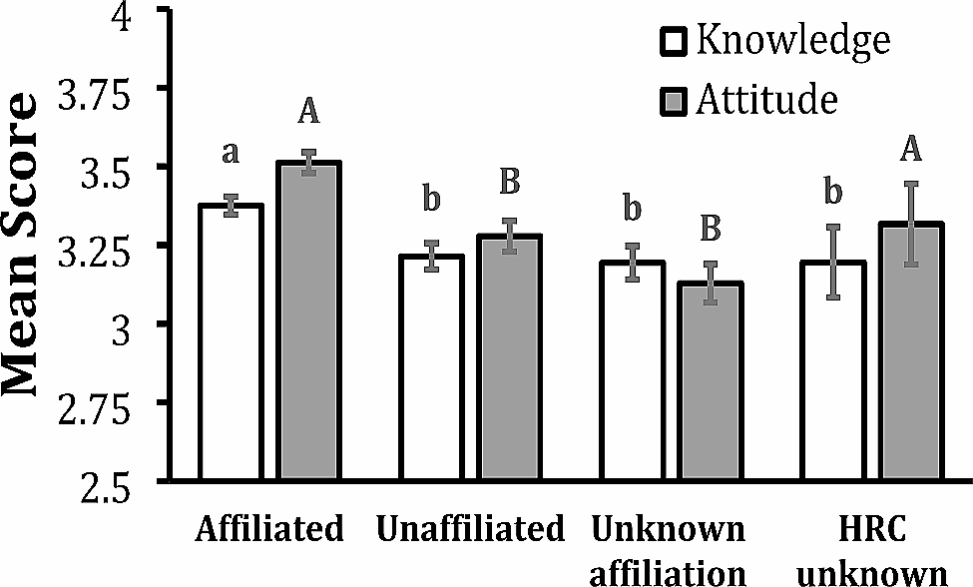
Mean Knowledge and Mean Attitude Scores by Harm Reduction Center (HRC) Affiliation. White columns represent mean knowledge scores ± SEM and gray columns represent mean attitude scores ± SEM. HRC unknown represents participants who selected “I do not know what a harm reduction center is.” Two knowledge means with the same lowercase letter are not significantly different at the 0.05 level. Note that providers practicing in a clinic or hospital affiliated with a harm reduction center had significantly higher mean knowledge scores than all other affiliation categories. Two attitude means with the same uppercase letter are not significantly different at the 0.05 level. Note that providers practicing in a clinic or hospital affiliated with a harm reduction center had higher mean attitude scores compared to providers working in unaffiliated environments or environments of unknown affiliation. There was no statistically significant difference in mean attitude scores between affiliated and HRC unknown. The overall p value for knowledge differences is *p* = 0.0014, and the overall *p* value for attitude differences is *p* < 0.0001

Respondents practicing at a facility affiliated with a harm reduction center (HRC) exhibited the highest mean knowledge and attitude scores compared to all the other response categories (Fig. 2). These differences were statistically significant across all categories for provider mean knowledge (*p* ≤ 0.01 and *p* < 0.0001, respectively). Mean attitude scores were significantly higher among providers practicing in a clinic or hospital affiliated with a HRC compared to providers working in unaffiliated environments or environments of unknown affiliation (*p* ≤ 0.05). There was no statistically significant difference in mean attitude scores between providers working in affiliated environments versus HRC unknown, which could be due to a smaller sample size in the HRC unknown category (*n* = 30, 4.3% of participants).

**Fig. 3:**
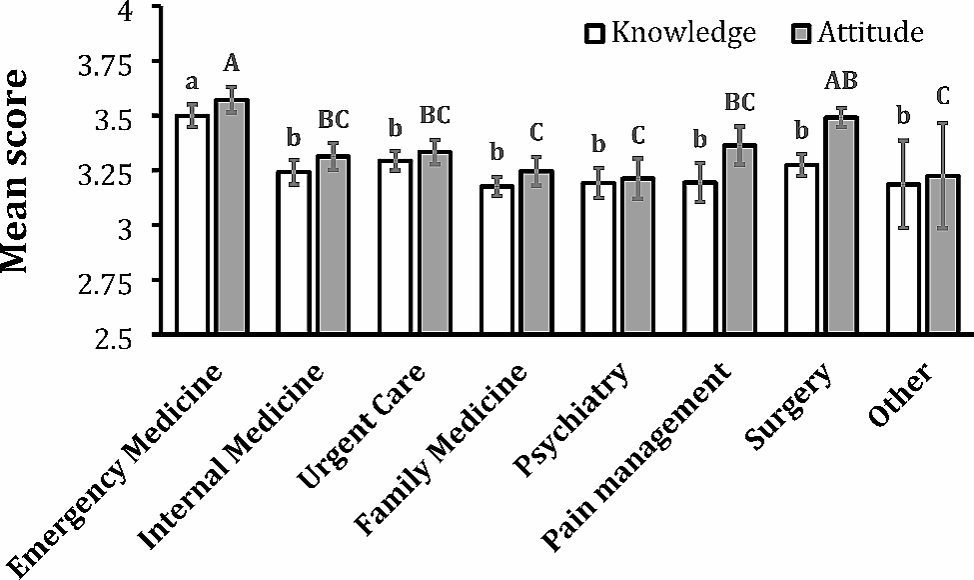
Mean Knowledge and Attitude Scores by Medical Specialty. White columns represent mean knowledge scores ± SEM and gray columns represent mean attitude scores ± SEM. Two knowledge means with the same lowercase letter are not significantly different at the 0.05 level. Note that emergency medicine providers had significantly higher mean knowledge scores than all other affiliation categories. Two attitude means with the same uppercase letter are not significantly different at the 0.05 level. Note that emergency medicine providers had significantly higher mean attitude scores compared to all other provider categories except surgery. Surgery providers had significantly higher mean attitude scores compared to family medicine, psychiatry, and the other category. The overall p value for knowledge differences is *p* < 0.0001, and the overall p value for attitude differences is *p* < 0.001

Among all the specialties, emergency medicine providers exhibited significantly higher mean knowledge scores (3.49) compared to all other specialties including internal medicine, urgent care, family medicine, psychiatry, pain management, surgery and an “other” category which ranged in mean scores from 3.17 to 3.29 (Fig. 3) (overall *p* < 0.001). Emergency medicine providers also had the highest attitude score (3.57), which was significantly greater than the attitude scores for all other subspecialties (3.21–3.36) except for surgery (3.49) (overall *p* < 0.001). Family medicine, psychiatry, and the “other” category had the lowest scores out of all the categories in both the knowledge (3.17, 3.19, and 3.18, respectively) and attitude (3.24, 3.21, and 3.22, respectively) sections.

**Fig. 4:**
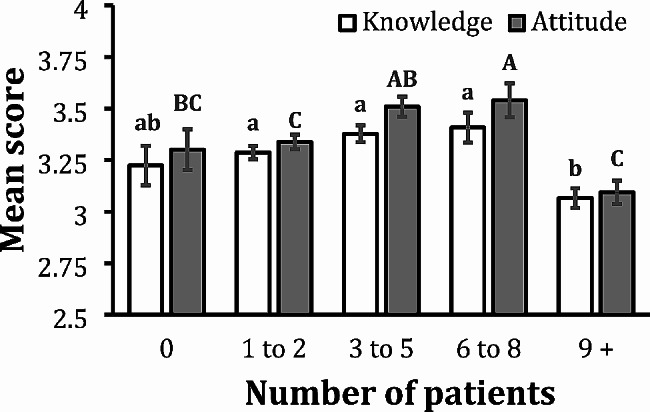
Mean Knowledge and Attitude Scores by Number of People Who Inject Drugs Encountered per Workday. White columns represent mean knowledge scores ± SEM and gray columns represent mean attitude scores ± SEM. Providers indicated their number of encounters with people who inject drugs (PWID) per typical workday as binned into discrete categories (0, 1-2, 3-5, 6-8, and 9+) on the survey. Two knowledge means with the same lowercase letter are not significantly different at the 0.05 level. Note that providers who encountered 9+ PWID during a typical workday had significantly lower mean knowledge scores than all other categories except zero encounters. Two attitude means with the same uppercase letter are not significantly different at the 0.05 level. Note that providers who encountered 9+ PWID during a typical workday had significantly lower mean attitude scores compared to providers who encountered 3-5 or 6-8 PWID in a typical workday. The overall p value for knowledge differences is *p* < 0.001, and the overall *p* value for attitude differences is *p* < 0.0001

The analysis of the number of encounters with PWID in a typical workday revealed an increasing trend in knowledge and attitude scores as the number of encounters increased across the discrete options of 0, 1–2, 3–5, and 6–8 encounters. This trend suddenly halted for respondents who reported nine or more encounters in a day, resulting in a significant drop in knowledge and attitude score (Fig. 4). Providers who interacted with nine or more PWID in a typical workday demonstrated the lowest knowledge score (3.06), which was significantly lower than knowledge scores for all other categories (mean score range: 3.28–3.40) except zero encounters (mean score 3.22) with PWID (overall *p* < 0.001). Providers who interacted with nine or more PWID in a typical workday also exhibited the lowest mean attitude score (3.09), which was significantly lower than the mean attitude scores for providers who encountered 3–5 or 6–8 PWID (3.50 and 3.54, respectively) but not significantly different from the mean attitude scores for providers who encountered 0 or 1–2 PWID (3.30 and 3.33, respectively).We constructed a contingency table to explore factors that might contribute to lower knowledge and attitudes among providers who encounter nine or more PWID per workday. Specifically, we tested the hypothesis that, among providers who encounter nine or more PWID per workday, the distribution of providers by specialty/department or by HRC affiliation is not significantly different than the distribution of providers who encounter < 9 PWID per workday among the same groups. In fact, the comparison by specialty/department was not significant (*p* = 0.4009) but the comparison by HRC affiliation was significant (*p* < 0.0001). Notably, the majority of providers (58.4%) who see less than 9 PWID per workday know they are affiliated with an HRC. In contrast, a plurality of providers (49.4%) who see more than 9 PWID per workday are unsure if their institution is affiliated with an HRC.

The vast majority (96.4%) of survey respondents were between the ages of 25 and 55 (Supplemental Table S3). No single age range category had a significantly higher or lower mean knowledge score than other age ranges. However, the 56–65 age range had the lowest mean knowledge score at 3.07 while the 66 + age range had the highest mean knowledge score at 3.69 (Supplemental Figure S2). Mean knowledge scores for the other age categories ranged from 3.25 to 3.36. Participants aged 56 to 65 years old exhibited a significantly lower mean attitude score (3.01; *p* < 0.05) than all the other age groups (mean score range: 3.36–3.68). The sample sizes for the 56–65 and 66 + age categories were small (19 and 6 participants, respectively) compared to the sample sizes (130–312) in other age categories, as indicated by the larger standard error of the means for the 56–65 and 66 + categories.

## Discussion

This research aimed to discover healthcare providers’ baseline knowledge about and attitudes towards OPS. The results from our study establish a strong correlation between evidence-based knowledge about OPS and a positive attitude about OPS. These findings indicate that a deficit in providers’ knowledge about OPS correlates with a more negative attitude toward OPS.

Healthcare providers can impact implementation of harm reduction strategies. For example, emergency physicians and pharmacists were evaluated on their perspectives about prescribing take-home naloxone, a drug that rapidly reverses opioid overdose, and found that the majority supported it, but had concerns. One of the most prominent concerns was not having sufficient education on naloxone [26, 29]. Data from our study suggest healthcare providers may play critical roles in advocacy and implementation of harm reduction strategies, like OPS.

Our study revealed a higher knowledge and attitude score among providers affiliated with HRCs, emphasizing the potential value of exposure to harm reduction strategies in shaping healthcare professionals’ perspectives. This finding suggests that increasing collaboration between HRCs and clinical centers could potentially improve providers’ understanding and attitudes towards OPS. In 2018, Canada had a substantial increase in patients presenting with infective endocarditis secondary to injection drug use. A three-step research strategy was implemented to integrate harm reduction at an acute care facility. First, healthcare personnel were educated about harm reduction. Second, harm reduction was explicitly supported in the facility. Third, providers were given access to harm reduction tools including consultations with addiction treatment programs as well as harm reduction kits that were supplied to eligible patients with the intent of being a conversation starter. Over 300 staff members participated in the educational sessions and self-reported an increase in knowledge surrounding harm reduction. Success of the harm reduction tools was measured by the length of a patient’s hospital stay for treatment of infective endocarditis. This three-part harm reduction approach resulted in longer hospital stays for patients and an increase in completion of treatment of infective endocarditis. The study authors concluded that patients felt the hospital was a safe place for treatment [28]. Much like our findings, this study reinforces the need for equipping healthcare providers with knowledge on harm reduction strategies to facilitate successful implementation.

Among our study participants, emergency medicine clearly exhibited the highest mean knowledge and attitude scores, further confirming our hypothesis that a more knowledgeable provider will have a more positive attitude about OPS. Contradictory to our hypothesis, the surgery specialty category exhibited the second highest mean attitude scores but relatively lower mean knowledge scores. This finding warrants further investigation to determine why surgeons have a more positive attitude about OPS despite an average knowledge base.

The significantly higher scores in knowledge and attitude among emergency medicine providers compared to the other specialties could be attributed to their frontline experience in dealing with opioid-related issues [29]. In 2014, Rhode Island’s Department of Health identified the emergency department as a key area in the implementation of harm reduction tools, including take home naloxone, as they were seeing approximately 40–50 overdoses per month [30]. Another study conducted in Boston, Massachusetts between 2011 and 2012 implemented an opioid overdose prevention protocol in the emergency department. Patients identified as being high risk for overdose were provided with overdose education only or overdose education and naloxone distribution. Although their study was limited due to low follow-up, they found patients who received naloxone kits had higher rates of administering naloxone, calling 911, and staying with the victim [31]. A 2019 mixed-methods study surveyed emergency department physician attitudes about initiation of opioid agonists treatment, specifically buprenorphine, in the emergency setting. Although the vast majority (80%) of physicians surveyed agreed that buprenorphine should be initiated for patients requesting it, only 44% felt comfortable discussing it. Follow-up interviews revealed that emergency providers may have responded negatively to PWUD due to their own frustrations with effectively employing treatment for opioid use disorder [32]. At an urban, safety-net hospital in California, implementation of a harm reduction program that educated patients and providers reduced addiction-related stigma and facilitated distribution of harm reduction kits [33]. Together with our results, these data identify the emergency department as a critical area for implementation of institutional processes in harm reduction strategies and a promising avenue for further research.

The relationship between the number of encounters with PWID in a typical workday and knowledge and attitude scores could suggest that increased exposure to PWID positively impacts providers’ perspective. However, the significance of this finding is limited by the sudden drop in scores among providers encountering nine or more PWID daily. Burnout and compassion fatigue among emergency responders may contribute to hostility towards PWID, particularly in under-resourced areas [34]. In this study, comparison of providers encountering nine or more PWID per workday to those who encounter less than 9 PWID per workday revealed no significant differences in the distribution of providers by specialty. The same comparison by HRC affiliation revealed that a plurality of providers (49.4%) who see nine or more PWID per workday are unsure if their institution is affiliated with an HRC. Given our finding of higher knowledge and attitude scores among providers affiliated with HRCs, these data reinforce the potential value of increasing collaboration between HRCs and clinical centers as a means to improve providers’ understanding of and attitudes towards OPS. Further research is necessary to identify the specific factors that contribute to significantly lower mean knowledge and attitude scores among providers working with a higher volume of PWID and to develop targeted interventions to help them care for PWID.

## Conclusions

Since 2012, there has been nearly a 50% decrease in opioid prescriptions nationally and greater than 200% increase in naloxone dispensed, yet the number of overdose deaths continues to rise with 109,360 overdose deaths in 2022 [24]. There is a need for implementation of additional harm reduction strategies. This research demonstrated that knowledge about OPS positively correlates with healthcare providers’ perceptions of OPS. Furthermore, providers who work in clinical environments associated with HRCs exhibit a more favorable attitude as well as greater knowledge towards OPS. Our study highlights the importance of education, exposure to harm reduction strategies, and inter-specialty collaboration in shaping healthcare providers’ knowledge and attitudes about OPS. Further research is needed to explore the factors underlying the observed trends, and targeted interventions should be developed to improve providers’ understanding and support for harm reduction strategies.

Although our study was limited in scope to providers in the state of Colorado, the large sample includes a diverse array of practice settings, provider roles, and specialties. The data presented suggest that dissemination of evidence-based information about OPS will positively impact providers’ attitudes and ultimately their support for OPS. Recent approval of a state-authorized OPS in Rhode Island and favorable data from New York indicate that OPS are gaining traction as a harm reduction strategy in the U.S. Working at federal, state, and local levels to provide healthcare professionals with more education about OPS may help save more lives by fueling advocacy for and implementation of OPS and other proven harm reduction tools.

### Electronic supplementary material

Below is the link to the electronic supplementary material.

Supplementary Material 1

## Data Availability

The datasets used and/or analyzed during the current study are available from the corresponding author on reasonable request.

## Abbreviations

HRC: Harm reduction center
NYC: New York City
OPS: Overdose prevention site(s)
PWID: People who inject drugs
PWUD: People who use drugs
RI: Rhode Island
U.S.: United States

## References

[1] National Center for Drug Abuse Statistics. Drug overdose death rates. https://drugabusestatistics.org/drug-overdose-deaths/. Accessed 7 February 2024.

[2] Ahmad FB, Cisewski JA, Rossen LM, Sutton P, February. National Center for Health Statistics. Provisional drug overdose death counts. https://www.cdc.gov/nchs/nvss/vsrr/drug-overdose-data.htm. Accessed 7 2024.

[3] Colorado Health Institute. A parallel epidemic: more overdose deaths in 2020, fentanyl fatalities spike. https://www.coloradohealthinstitute.org/research/2020overdose_dashboard. Accessed 17 October 2023.

[4] Drug Policy Alliance. Overdose Prevention Centers (OPCs). https://drugpolicy.org/issue/overdose-prevention-centers-opcs/. Accessed 16 October 2023.

[5] Potier C, Laprévote V, Dubois-Arber F, Cottencin O, Rolland B. Supervised injection services: what has been demonstrated? A systematic literature review. Drug Alcohol Depend. 2014. 10.1016/j.drugalcdep.2014.10.012. 25456324 10.1016/j.drugalcdep.2014.10.012

[6] OnPoint NYC. https://onpointnyc.org/. (2021). Accessed 17 March 2024.

[7] Folch C, Lorente N, Majó X, et al. Drug consumption rooms in Catalonia: a comprehensive evaluation of social, health and harm reduction benefits. Int J Drug Policy. 2018. 10.1016/j.drugpo.2018.09.008. 30352331 10.1016/j.drugpo.2018.09.008

[8] Kerr T, Stoltz JA, Tyndall M, et al. Impact of a medically supervised safer injection facility on community drug use patterns: a before and after study. BMJ. 2006;332(7535):220–2.10.1136/bmj.332.7535.220 16439401 10.1136/bmj.332.7535.220 PMC1352057

[9] Kennedy MC, Karamouzian M, Kerr T. Public health and public order outcomes associated with supervised drug consumption facilities: a systematic review. Curr HIV/AIDS Rep. 2017. 10.1007/s11904-017-0363-y. 28875422 10.1007/s11904-017-0363-y

[10] Freeman K, Jones CGA, Weatherburn DJ, Rutter S, Spooner CJ, Donnelly N. The impact of the Sydney medically supervised injecting centre (MSIC) on crime. Drug Alcohol Rev. 2005. 10.1080/09595230500167460. 16076587 10.1080/09595230500167460

[11] Fitzgerald J, Burgess M, Snowball L, NSW Bureau of Crime and Statistics and Research. Trends in Property and Illicit drug crime around the Medically Supervised Injecting Centre in Kings Cross: An Update. 2010. https://www.bocsar.nsw.gov.au/Publications/BB/bb51.pdf. Accessed 10 December 2023.

[12] Irwin A, Jozaghi E, Bluthenthal RN, Kral AH. A cost-benefit analysis of a potential supervised injection facility in San Francisco, California, USA. J Drug Issues. 2017. 10.1177/0022042616679829. 10.1177/0022042616679829

[13] Giglio RE, Mantha S, Harocopos A, et al. The nation’s first publicly recognized overdose prevention centers: lessons learned in New York City. J Urban Health Bull N Y Acad Med. 2023. 10.1007/s11524-023-00717-y. 10.1007/s11524-023-00717-y PMC10072795 37016269

[14] Rhode Island Department of State. Harm reduction centers (216-RICR-40-10-25). https://rules.sos.ri.gov/regulations/part/216-40-10-25. Accessed 30 October 2023.

[15] Project Weber/RENEW. The country’s first state-regulated overdose prevention center. https://weberrenew.org/overdose-prevention-center/ (2024). Accessed January 5 2024.

[16] National Institutes of Health. A comparative evaluation of overdose prevention programs in New York City and Rhode Island. 2023. https://reporter.nih.gov/search/ilOT4ZmcrkO6TOGMLAKkig/project-details/10629749. Accessed October 17 2023.

[17] Chalfin A, Del Pozo B, Mitre-Becerril D. Overdose prevention centers, crime, and disorder in New York City. JAMA Netw Open. 2023. 10.1001/jamanetworkopen.2023.42228. 37955901 10.1001/jamanetworkopen.2023.42228 PMC10644216

[18] Colorado General Assembly. Overdose prevention center authorization. 2023. https://leg.colorado.gov/bills/hb23-1202. Accessed 15 December 2023.

[19] Colorado General Assembly. Substance Use Disorder Harm Reduction. 2018. https://leg.colorado.gov/bills/sb18-040. Accessed 15 December 2023.

[20] Kral AH, Lambdin BH, Wenger LD, Davidson PJ. Evaluation of an unsanctioned safe consumption site in the United States. N Engl J Med. 2020. 10.1056/NEJMc2015435. 32640126 10.1056/NEJMc2015435

[21] Armbrecht E, Guzauskas G, Hansen R et al. Institute for Clinical and Economic Review. Supervised injection facilities and other supervised consumption sites: effectiveness and value; final evidence report. 2021. https://icer.org/wp-content/uploads/2020/10/ICER_SIF_Final-Evidence-Report_010821.pdf. Accessed 28 December 2023.

[22] Samuels EA, Bailer DA, Yolken A. Overdose prevention centers: an essential strategy to address the overdose crisis. JAMA Netw Open. 2022. 10.1001/jamanetworkopen.2022.22153. 35838675 10.1001/jamanetworkopen.2022.22153

[23] American Medical Association. AMA wants new approaches to combat synthetic and injectable drugs. 2017. https://www.ama-assn.org/press-center/press-releases/ama-wants-new-approaches-combat-synthetic-and-injectable-drugs. Accessed 25 October 2023.

[24] American Medical Association. Overdose epidemic report 2023: physicians’ actions to help end the nation’s drug-related overdose and death epidemic—and what still needs to be done. https://www.ama-assn.org/system/files/ama-overdose-epidemic-report.pdf. Accessed 16 November 2023.

[25] Harm Reduction Action Center. Health providers. 2020. https://www.harmreductionactioncenter.org/health-providers. Accessed 17 October 2023.

[26] Holland TJ, Penm J, Dinh M, Aran S, Chaar B. Emergency department physicians’ and pharmacists’ perspectives on take-home naloxone. Drug Alcohol Rev. 2019. 10.1111/dar.12894. 30697852 10.1111/dar.12894

[27] Guy GP, Haegerich TM, Evans ME, Losby JL, Young R, Jones CM. Vital signs: pharmacy-based naloxone dispensing — United States, 2012–2018. Morb Mortal Wkly Rep. 2019. 10.15585/mmwr.mm6831e1. 10.15585/mmwr.mm6831e1 PMC6687198 31393863

[28] Hyde EK, Nguyen T, Gilchrist S, Lee-Ameduri K. Integrating harm reduction into acute care: a single center’s experience. JTCVS Open. 2023. 10.1016/j.xjon.2023.05.005. 37808025 10.1016/j.xjon.2023.05.005 PMC10556805

[29] Wilson HD, Dansie EJ, Kim MS, Moskovitz BL, Chow W, Turk DC. Clinicians’ attitudes and beliefs about opioids survey (CAOS): instrument development and results of a national physician survey. J Pain. 2013. 10.1016/j.jpain.2013.01.769. 23541067 10.1016/j.jpain.2013.01.769

[30] Samuels E. Emergency department naloxone distribution: a Rhode Island department of health, recovery community, and emergency department partnership to reduce opioid overdose deaths. RI Med J. 2014;97(10):38–9. 25271659

[31] Dwyer K, Walley A, Langlois B, et al. Opioid education and nasal naloxone rescue kits in the emergency department. West J Emerg Med. 2015. 10.5811/westjem.2015.2.24909. 25987910 10.5811/westjem.2015.2.24909 PMC4427207

[32] Im DD, Chary A, Condella AL, et al. Emergency Department clinicians’ attitudes toward Opioid Use Disorder and Emergency Department-initiated Buprenorphine Treatment: a mixed-methods study. West J Emerg Med. 2020;21(2):261–71. 10.5811/westjem.2019.11.44382. Published 2020 Feb 21. 32191184 10.5811/westjem.2019.11.44382 PMC7081867

[33] Perera R, Stephan L, Appa A, et al. Meeting people where they are: implementing hospital-based substance use harm reduction. Harm Reduct J. 2022;19:14. 10.1186/s12954-022-00594-9. 35139877 10.1186/s12954-022-00594-9 PMC8826677

[34] Ondocsin J, Mars SG, Howe M, Ciccarone D. Hostility, compassion and role reversal in West Virginia’s long opioid overdose emergency. Harm Reduct J. 2020;17(1):74. 10.1186/s12954-020-00416-w. 33046092 10.1186/s12954-020-00416-w PMC7549084

