# Supplementary material for: Healthcare providers’ knowledge and attitudes about overdose prevention sites in Colorado
Source: Harm Reduct J. 2024 Aug 24;21:155. doi: 10.1186/s12954-024-01066-y (PMC11344454; doi:10.1186/s12954-024-01066-y)
Supplement: Supplementary file 1 — Supplementary Material 1 [file 12954_2024_1066_MOESM1_ESM.docx]

SUPPLEMENTAL TABLES and FIGURES for:

Healthcare Providers’ Knowledge and Attitudes About Overdose Prevention Sites in Colorado

Emily Paz^1^, Vahid Mashhouri^1^, Mark E. Payton^2^, Brian D. Schwartz^3^, Rachel M.A. Linger^2^*

^1^College of Osteopathic Medicine, Rocky Vista University, Englewood, CO, United States

^2^Biomedical Sciences Department, Rocky Vista University, Englewood, CO, United States

^3^Medical Humanities Department, Rocky Vista University, Englewood, CO, United States

*Corresponding author: [rlinger@rvu.edu](mailto:rlinger@rvu.edu)

**Supplemental Table S1. Survey Statements**

| Demographic Information |  |  |
| --- | --- | --- |
| In which of the following settings do you predominantly practice?   - Rural - Urban - Suburban - Other: __________ | | |
| Is your hospital or clinic affiliated with a harm reduction center?   - Yes - No - I do not know if my hospital is affiliated - I do not know what a harm reduction center is | | |
| What is your role?   - MD - DO - NP - PA | | |
| What is your specialty/department?   - Emergency Medicine - Internal Medicine - Urgent care - Family Medicine - Psychiatry - Pain management - Surgery - Other: _______________ | | |
| How many patients who inject drugs (PWID) do you encounter in a typical workday?   - 0 - 1-2 - 3-5 - 6-8 - 9+ | | |
| What is your age range?   - 25-35 - 36-45 - 46-55 - 56-65 - 66+ | | |

**Supplemental Table S1. (continued)**

| Definitions |
| --- |
| There is a lack of uniformity in the industry as to the verbiage used for overdose prevention sites, including safe consumption sites, safe injection facilities, drug consumption rooms, etc. Because they all refer to the same type and purpose of facility, for the purposes of this survey, we will subsume each of those under the name of overdose prevention sites (OPS).  Abbreviations used in this survey:  OPS = Overdose Prevention Sites  PWID = People Who Inject Drugs |
| Knowledge Statements  To what extent do you agree with the following statements:  Likert scale for all statements:  Strongly disagree, Somewhat disagree, Neither agree nor disagree, Somewhat agree, Strongly agree |
| 1. It is important to keep current on the literature regarding OPS. |
| 1. OPS will encourage drug use. |
| 1. OPS are a cost-effective harm reduction strategy. |
| 1. Drug rehabilitation centers are more cost effective than OPS. |
| 1. OPS will increase the number of local drug users. |
| 1. OPS are an effective way to connect PWID with other services including healthcare. |
| 1. OPS are an effective way to decrease fatalities from drug overdose. |
| Attitude Statements  To what extent do you agree with the following statements:  Likert scale for all statements:  Strongly disagree, Somewhat disagree, Neither agree nor disagree, Somewhat agree, Strongly agree |
| 1. I am willing to advocate for OPS in the community. |
| 1. I would volunteer at an OPS. |
| 1. I have a responsibility to discuss harm reduction strategies with patients when applicable. |
| 1. I would recommend my patients utilize an OPS if they were available. |
| 1. OPS violate my duty to do no harm. |
| 1. OPS promote harmful health behaviors like injection drug use. |
| 1. OPS are beneficial to PWID. |
| 1. OPS are beneficial to the community in which they are located. |

**Supplemental Table S2. Scoring Key for Survey Questions**

|  | Knowledge Score | | Attitude Score | |
| --- | --- | --- | --- | --- |
| Response | **Statements***  **1, 3, 6-7** | **Statements***  **2, 4-5** | **Statements***  **8-11, 14-15** | **Statements***  **12-13** |
| Strongly disagree | 1 | 5 | 1 | 5 |
| Somewhat disagree | 2 | 4 | 2 | 4 |
| Neither agree nor disagree | 3 | 3 | 3 | 3 |
| Somewhat agree | 4 | 2 | 4 | 2 |
| Strongly agree | 5 | 1 | 5 | 1 |

* See Supplemental Table S1 for identification of individual statements.

**Supplemental Table S3.**

**Participant demographic data table**

| *Variable* |  |  |
| --- | --- | --- |
| *Practice Setting* | ***n*** | ***%*** |
| Rural  Suburban  Urban  Other | 119  85  476  15 | 17.1  12.2  68.5  2.2 |
| *HRC Affiliation* | ***n*** | ***%*** |
| Yes  No  I don’t know if affiliated  I don’t know what HRC is | 382  162  120  30 | 55.0  23.3  17.3  4.3 |
| *Provider Role* | ***n*** | ***%*** |
| MD  DO  PA  NP | 274  187  182  52 | 39.4  26.9  26.2  7.5 |
| *Specialty/Department* | ***n*** | ***%*** |
| Emergency Med  Internal Med  Urgent Care  Family Med  Psychiatry  Pain Management  Surgery  Other | 159  109  145  105  43  53  73  10 | 22.8  15.6  20.8  15.1  6.2  7.6  10.5  1.4 |
| *Daily PWID encounters* | ***n*** | ***%*** |
| 0  1-2  3-5  6-8  9+ | 44  274  213  82  83 | 6.3  39.4  30.6  11.8  11.9 |
| *Age* | ***n*** | ***%*** |
| 25-35  36-45  46-55  56-65  66+ | 228  312  130  19  6 | 32.8  44.9  18.7  2.7  0.9 |

**Supplemental Figure 1.**

**Supplemental Figure S1. Comparison of Mean Knowledge and Mean Attitude Scores by Provider Type.** White columns represent mean knowledge scores ± SEM and gray columns represent mean attitude scores ± SEM. No one provider role scored significantly higher or lower than the rest in both knowledge and attitude.

**Supplemental Figure 2.**

**Supplemental Figure S2. Mean Knowledge and Attitude Scores by Provider Age Group.** White columns represent mean knowledge scores ± SEM and gray columns represent mean attitude scores ± SEM. Providers indicated their age as binned into discrete categories (25-35, 36-45, 46-55, 56-65, and 66+) on the survey. There were no statistically significant differences in mean knowledge scores across age groups (overall *p* = 0.0613). Providers aged 56-65 exhibited the lowest mean knowledge and attitude compared to all other age categories, with the mean attitude score being significantly lower compared to all other age groups (asterisk). The overall *p* value for attitude differences is *p* < 0.05.
